# Performance evaluation of three commercial molecular assays for the detection of *Mycobacterium tuberculosis* from clinical specimens in a high TB-HIV-burden setting

**DOI:** 10.1186/s12879-015-1229-9

**Published:** 2015-11-09

**Authors:** M. M. Z. Matabane, F. Ismail, K. A. Strydom, O. Onwuegbuna, S. V. Omar, N. Ismail

**Affiliations:** Department of Medical Microbiology, University of Pretoria, Pretoria, South Africa; National Health Laboratory Services, Tshwane Academic Division, Pretoria, South Africa; National Institute for Communicable Diseases: Centre for Tuberculosis, Johannesburg, South Africa

**Keywords:** GenoType MTBDR*plus*, Xpert® MTB/RIF, Anyplex™ plus, *Mycobacterium tuberculosis*, Detection, Molecular assays, TB-HIV, South Africa

## Abstract

**Background:**

A major challenge faced by countries with a high burden of tuberculosis (TB) is early detection especially in individuals with paucibacillary disease which is common in HIV endemic settings.

Remarkable efforts have been made globally to accelerate the development and expansion of new diagnostic technologies that allow better and earlier diagnosis of active tuberculosis particularly directly from clinical specimens with a few commercial options available. These include GenoType MTBDR*plus* Version 2.0 (Hain Lifescience), Xpert® MTB/RIF (Cepheid) and Anyplex™ plus MTB/NTM/DR-TB Real-time detection (Seegene).

We evaluated the diagnostic performance of these three commercial molecular assays for the detection of *Mycobacterium tuberculosis* complex from clinical specimens in a high TB-HIV-burden setting.

**Methods:**

This was a retrospective laboratory-based study using stored remnant sediments from clinical specimens of presumptive pulmonary TB cases. A stratified sample of smear positive TB, smear negative TB and TB culture negatives was included. All the samples were tested on the three molecular assays following the manufacturers’ instructions; except for Anyplex™plus, for which DNA extraction was performed using the NucliSENS® easyMAG® platform (bioMerieux). Samples were also processed for liquid TB culture and time-to-culture positivity was recorded.

**Results:**

Of the 90 sediments processed, 81 were analyzable across all three systems. The overall sensitivity was highest for Xpert® MTB/RIF (89.1 %) followed by GenoType MTBDR*plus* (70.9 %) and Anyplex™ plus (65.5 %). The specificity and sensitivity in smear positive cases was comparable across all systems. There was a significant difference in sensitivity between Xpert® MTB/RIF and the other two assays for smear-negative cases (*P* < 0.05). The performance in cases where the time-to-culture positivity was ≥20 days was also significantly poorer for both Anyplex™ plus and GenoType MTBDR*plus* compared to Xpert® MTB/RIF (*P* < 0.05). Xpert® MTB/RIF achieved 100 % specificity, while Anyplex™ plus and GenoType MTBDR*plus* achieved 96.2 and 92.3 % respectively.

**Conclusion:**

The Xpert® MTB/RIF was superior to the other two assays for the detection of TB in smear negative specimens notably when bacterial loads are very low in sputum. It is important that studies reporting on test performance stratify their results by time-to-culture positivity to accurately assess clinical performance especially in high HIV settings.

## Background

Tuberculosis (TB) has plagued humankind for thousands of years [1]. It still remains a major global public health challenge despite being declared by the World Health Organization (WHO) more than twenty years ago, as a public health emergency [2]. In 2012, the case detection rate was 66 % worldwide, which is below the minimum 70 % target [2]. One of the major reasons for this short-fall is the lack of accurate and reliable diagnostic tools [3].

In most resource-limited countries, conventional smear microscopy is the most widely used primary laboratory method for TB diagnosis [4]. Although cheap and relatively easy to perform; this century old method has variable sensitivity rates ranging from 32 to 94 % [5]. The sensitivity often depends on the diligence with which the specimens are collected and processed [4]. The modification of smear microscopy, which includes, physical and chemical processing methods and the use of fluorescence microscopy has improved sensitivity with specificity remaining comparable [5, 6]. This is especially relevant among children and people living with human immunodeficiency virus (HIV) with smear-negative pulmonary TB [5–7].

Over the years, there has been significant advancement in molecular technologies, particularly development of real-time polymerase chain reaction (PCR) testing platforms. The main advantages of real-time PCR are shortened turn-around-time, automation of the procedure which reduces hands-on time and risk of cross-contamination [3, 4, 8]. There are currently two WHO-endorsed molecular technologies, namely, GenoType MTBDR*plus* (Hain Lifescience GmbH, Nehren, Germany) and Xpert® MTB/RIF (Cepheid, USA) [2]. New molecular tests are increasingly being developed for the diagnosis of TB, thus, TB programs and diagnostic laboratories are often faced with the dilemma of choosing between the molecular options available.

GenoType MTBDR assay (Hain Lifescience GmbH, Nehren, Germany) was introduced in 2004 and is a PCR amplification and reverse hybridization assay that simultaneously detects *M. tuberculosis* complex (MTBC) as well as mutations in the *rpoB* gene for rifampicin resistance and the *katG* gene for high-level isoniazid resistance [9]. The second generation assay, GenoType MTBDR*plus* detects in addition mutations in the *inhA* gene that confer low-level isoniazid resistance [9, 10]. GenoType MTBDR*plus* version 1.0 has been proven to be robust and accurate but limited to smear-positive or culture positive isolates [10]. The assay was endorsed by the WHO in 2008, with the intention to enhance capacity for rapid diagnosis of drug resistant tuberculosis and reviewed in a meta-analysis by Ling and colleagues [2, 9]. GenoType MTBDR*plus* version 2.0 has been released for use in smear-negative specimens, which provides an opportunity to exclude the culture step thus reducing the turnaround time and has demonstrated a sensitivity of 56.6 % in smear-negative TB cases in the South African setting [11].

The Xpert® MTB/RIF endorsed by WHO in 2010, is an automated hemi-nested real-time PCR assay that simultaneously detects *Mycobacterium tuberculosis* complex and rifampicin resistance [12–14]. This is a simple assay that can be performed with minimal training and provides results within two hours, including a 15-minute manual sample preparation step [12–14]. Steingart et al. demonstrated a pooled sensitivity of 98 % for smear-positive cases and 67 % for smear-negative TB cases [12].

The Anyplex™ plus MTB/NTM/DR-TB (Seegene, Seoul, Republic of Korea) is a commercially available multiplex real-time PCR system but not endorsed by the WHO. The assay uses two technologies, namely, the Dual Priming Oligonucleotide (DPO™) technology and the Real Amplicon Detection (READ) technology [15]. These technologies enable the test to perform multi-target detection with high specificity and sensitivity [15]. The DPO™ technology provides freedom in primer design and PCR optimization and maximizes PCR sensitivity and specificity by blocking non-specific priming [15]. The READ technology allows high-throughput multiplexing onto a real-time PCR platform [15]. The assay is a two-step open system that detects MTB/NTM first. Drug susceptibility testing for MTBC is a second PCR reaction that is not continuous with the MTB/NTM detection step. Thus, amplicons generated in the MTB/NTM detection step need to be manually pipetted to a second PCR reaction for assessment of drug susceptibility. A comparison of the three molecular systems is shown in Table 1. To our knowledge, there have been no published studies evaluating the performance of Anyplex™ plus MTB/NTM/DR-TB in a high TB-HIV-burden setting.

**Table 1:** Comparison of operational characteristics the three molecular assays

	Anyplex™ plus MTB/NTM/DR-TB	GenoType MTBDR*plus* version 2.0	Xpert® MTB/RIF
Principle	Two-step multiplex real time PCR	Reverse hybridisation	Hemi-nested real-time PCR
End result	MTBC, NTM, RIF, INH	MTBC, RIF, INH	MTBC, RIF
Level of complexity	High	High	Low
Turn-around-time (including DNA extraction)	3.5 h	5.35 h	2 h
Result interpretation	Automated	Manual^a^	Automated
Throughput per run	96	48	16^b^
Laboratory infrastructure	Open system requiring three separate work areas.	Open system requiring three separate work areas.	Closed system, bench top within a temperature controlled area

^a^Genoscan required for automated interpretation of results but at an additional cost

^b^Depends on which module you have (e.g. 4, 16, or 48)

We evaluated the diagnostic performance of these three commercial molecular assays for the detection of *Mycobacterium tuberculosis* complex (MTBC) from clinical specimens in a high TB-HIV-burden setting.

## Methods

This study was conducted at the National Health Laboratory Service (NHLS) diagnostic microbiology laboratory (TB section), Tshwane Academic Division, Pretoria, South Africa. This is an ISO 15189 accredited laboratory at a tertiary academic institution and provides TB diagnostic services to tertiary and district hospitals, and surrounding primary health care clinics. This was a laboratory based retrospective study which did not have prior informed patient consent when samples were routinely collected for TB investigations, and it was not feasible to acquire the consent retrospectively. We requested and received a waiver of informed consent from the Health Research Ethics Committee at the University of Pretoria, which is an accredited ethics committee (Ethics Reference No.: 445/2013).

Remnant sediments from presumptive pulmonary TB cases submitted for TB investigations, which included fluorescent TB microscopy and culture were retrieved from the departmental storage section; stored at 2–8 °C for a maximum of 3 months. Sediments with at least 3.5 ml volume were included, and we selected sequential culture positive and culture negative sediments in a 2:1 ratio with a total sample size of 90 sediments; 60 culture positive and 30 culture negative.

These sediments were from respiratory samples processed following the laboratory’s standard operating procedures. Briefly, after digestion with dithiotreitol-containing digestant and centrifugation at 3000 × g for 20 min, an aliquot of the sediment was subjected to smear microscopy using the auramine-O fluorescence stain. The smears were graded according to the International Union Against Tuberculosis and Lung Disease (IUATLD) guidelines. All the digested samples were then decontaminated with initial concentration of 4 % sodium hydroxide solution and allowed to stand for 25 min at room temperature. The phosphate buffer was then added to dilute the mixture and centrifuged at 3000 × g for 20 min. The remnant sediments were split into three aliquots of 1 ml each and randomly assigned to one of the three assays to minimize bias. A 0.5 ml aliquot was inoculated into the BACTEC™ MGIT™ 960 Mycobacterial Detection System (BD Diagnostics, USA) to reconfirm the culture results and incubated for a maximum of 42 days. Positive cultures were confirmed to be mycobacteria by performing Ziehl-Neelsen (ZN) staining, and as MTBC using the TB Ag MPT64 Rapid (SD Bioline, South Korea). If the ZN was negative for acid fast bacilli, this was regarded as a contaminated culture. No further re-decontamination was attempted on any of the positive MGIT cultures. Time-to-culture-positivity (TTP) of the primary culture was recorded and regarded as final.

Testing on the three molecular platforms, namely, Anyplex™ plus MTB/NTM/DR-TB Real-time detection (Anyplex™plus), GenoType MTBDR*plus* version 2.0 (MTBDR*plus*) with GenoLyse® extraction, and Xpert® MTB/RIF (Xpert®) followed the manufacturers’ instructions; except for extraction of Anyplex™plus. For the latter, DNA extraction was performed using the NucliSENS® easyMAG® platform (bioMerieux, France), using the generic protocol, as initial preparatory work indicated that the extraction procedure as prescribed by the manufacturer was not optimal resulting in PCR inhibition. Invalid results on any of the three assays were repeated and excluded if they remained invalid.

All the results were tabulated according to smear and culture status. Sensitivity was calculated as proportion of positives over the true positives for each stratum (scanty positive, smear positive and negative). Specificity was calculated as a proportion of negatives over true negatives. Additionally, the sensitivity calculation was performed on smear negative sediments for those with a TTP of less than 20 and 20 days or more. A McNemar test for symmetry was used to assess significant differences between the performance of each test and a *P* value of ≤0.05 was considered significant. Bivariate correlation was performed comparing TTP and Xpert® cycle threshold (Ct) score for the smear negative TB cases.

## Results

Of the 90 sediments processed, nine were excluded from analysis because of culture contamination (8) or invalid result (1), thus 81 results were analyzable across all three systems resulting in 55/60 (91.7 %) culture positive and 26/30 (86.7 %) culture negative. Of the culture positive, 29/55 (52.7 %) were smear positive; six being scanty positive and the remaining 26 smear negative cases.

The overall sensitivity was highest for Xpert® at 89.1 % followed by MTBDR*plus* at 70.9 % and Anyplex™ plus at 65.5 % (Table 2). The specificity across all systems was comparable with Xpert® achieving the highest specificity (100 %). Among the smear positive cases, Anyplex™ plus unlike the other two systems had false negative results for 2/23 cases.

**Table 2:** Performance of the molecular assays compared to culture

Category	Anyplex™ plus MTB/NTM/DR-TB	GenoType MTBDR*plus* version 2.0	Xpert® MTB/RIF
Sensitivity (culture positive for MTB)			
Overall sensitivity (*n* = 55)	36 (65.5 %)	39 (70.9 %)	49 (89.1 %)
Smear Positive (*n* = 23)	21 (91.3 %)	23 (100 %)	23 (100 %)
Scanty Positive (*n* = 6)	3 (50 %)	4 (66.7)	5 (83.3 %)
Smear negative (*n* = 26)	12 (46.2 %)	12 (46.2 %)	21 (80.8 %)*
Specificity			
Culture negative (*n* = 26)	25 (96.2 %)	24 (92.3 %)	26 (100 %)

*P < 0.05 when Xpert® is compared to Anyplex™ plus and when Xpert® is compared to MTBDR*plus*

There was a significant difference in sensitivity between Xpert® and the other two assays for smear-negative cases (*P* < 0.05). Among the smear negative cases, 12 were culture positive within 20 days and 14 were positive at day 20 or later. The performance of smear-negative cases was 75 % or higher in isolates with a TTP of less than 20 days across all systems with no significant difference (*P* = 0.5) (Table 3). However, the performance in cases where the TTP was ≥20 days was significantly poorer for both Anyplex™ plus and MTBDR*plus* compared to Xpert® (*P* < 0.05) (Table 3). There was no correlation (*r* = 0.08) between the Xpert Ct scores and the TTP among the smear negative TB cases (Table 4).

**Table 3:** Sensitivities of the molecular assays based on Time-To-Culture positivity for smear negative TB specimens

Time-to-CulturePositivity	Anyplex™ plus MTB/NTM/DR-TB	GenoType MTBDR*plus* version 2.0	Xpert® MTB/RIF
<20 days (*n* = 12)	9 (75 %)	9 (75 %)	11 (91.2 %)
≥20 days (*n* = 14)	3 (21 %)	3 (21 %)	10 (71 %)*

*P < 0.05 when Xpert® is compared to Anyplex™ plus and when Xpert® is compared to MTBDR*plus*

**Table 4:** Comparison of Xpert® Ct scores and Time-To-Culture Positivity (TTP)

Study Number	TTP	Probe D	Probe C	Probe E	Probe B	Probe A	Xpert® interpretation	Ave Ct
TTP <20d								
138	11	20.8	19.3	20.8	21.3	19.2	Medium	20.28
122	14	20.9	19	20	22.3	18.6	Medium	20.16
127	14	14.7	13.3	15.2	14.9	13.2	High	14.22
130	14	16.5	15.1	16.5	16.6	14.9	High	15.92
132	14	20.7	19.2	20.6	20.3	19	Medium	19.96
142	14	17.9	16.6	18.1	0	16.5	Medium	13.82
144	15	26.3	25.0	26.3	26.3	24.7	Low	25.72
147	15	26.7	25.5	27.1	26.6	25.4	Low	26.26
131	16	28.1	26.6	28.2	28.1	26.6	Low	27.52
139	16	34	33	34.8	33.3	33	Low	33.62
121	18	32.9	31.8	33.7	32.2	32.1	Very Low	32.54
145	19	Not detected						
TTP ≥20d								
133	20	23.2	21.7	23.2	23.2	21.6	Medium	22.58
136	21	Not detected						
146	21	26.9	25.4	26.9	26.9	25.4	Low	26.3
120	21	20.4	18.8	20.1	20.3	18.7	Medium	19.66
124	21	35.1	33.8	36.1	34.3	34.1	Very Low	34.68
125	21	33.7	32.5	34.5	33.0	32.6	Very Low	33.26
126	21	Not detected						
137	21	Not detected						
128	22	27.3	25.9	27.3	27.4	25.8	Low	26.74
129	22	23.6	22.1	23.6	23.5	22.0	Medium	26.74
134	22	21.3	19.7	21.2	21.2	19.5	Medium	20.58
119	23	26.9	25.8	27	26.7	25.6	Low	26.4
135	26	24.1	22.8	24.1	24.1	22.5	Low	23.52
123	30	Not Detected						

## Discussion

This study was the first to directly compare these three commercially available assays in a high HIV and TB prevalence setting and demonstrated that Xpert® had the best overall performance for the detection of TB from clinical specimens. The Xpert®, MTBDR*plus* and Anyplex™ plus showed overall sensitivities of 89.1 %, 70.9 and 65.5 % respectively. The performance of the Xpert® MTB/RIF in our study is comparable with a meta-analysis by Steingart et al. showing a pooled sensitivity of 98 % for smear positive cases, however, for the smear negative cases the sensitivity in our study was higher (80 % vs 67 %) [12]. Although our study showed comparable overall sensitivity for Xpert® with published literature; it was not the case for the other two assays. However, when restricted to TTP of less than 20 days, their performances are comparable to those in the literature. All three assays failed to detect six (10.9 %) culture-positive TB cases; five smear-negative and one scanty-positive which influenced their overall sensitivities. These isolates had a mean TTP of 23 days (range 19–30 days) suggesting that the number of bacilli in these specimens was low.

Time-to-liquid culture positivity has been shown to be an alternative measure of bacterial burden and has an inverse correlation with the number of colony forming units on solid media and bacilli on microscopy [16, 17]. An important finding in our study was the significant differences in sensitivity rates of smear negative cases with TTP of 20 days or longer. This was not observed in smear positive cases or smear negative cases with TTP of less than 20 days which indicates that the superior performance of Xpert® for TB detection is due to its ability to detect low bacillary loads compared to the other assays and further corroborated by the low level of detection of the assay. However, when the average Ct scores in the smear negative group were compared between those with a TTP <20 days to those with a TTP ≥20 days there was no correlation. This could be explained by the fact that the Xpert® system, has a filter capture system that concentrates *M. tuberculosis* bacilli and is a hemi-nested real time assay which aims to increases test sensitivity but this 2 stage cycling approach means that the Ct scores reported may not directly relate to the original bacterial load. This methodology would be especially true when the original bacterial load is low. Furthermore, Omar et al. showed no correlation between mean Ct scores for concentrations of *M. tuberculosis* between 25 and 2500 CFU/ml, which relates to smear negative cases [18].

MTBDR*plus* has been evaluated for diagnosis of smear-negative TB cases and has had variable performance rates in different settings. Crudu et al. demonstrated a sensitivity rate of 76 % when MTBDR*plus* was compared to culture as the gold standard in a setting with an estimated 4.7 % prevalence of TB/HIV co-infection in 2009 [19]. Barnard et al. reported 56.6 % detection rate of smear-negative TB cases in a high TB and HIV prevalence setting [11]. In our study with an even high rate of HIV population prevalence than the Barnard paper (12.4 % compared with 5 %), we reported an overall sensitivity of 46.2 % which is lower compared to the other studies [20]. However, when restricted to TTP of less than 20 days, sensitivity is comparable with that demonstrated by Crudu et al. [19]. Our study possibly explains the performance variability of MTBDR*plus* in different settings and patient population differences especially related to the relative risk of paucibacillary disease in patients being sampled. The inconsistency in the performance of MTBDR*plus* for smear-negative TB cases in different settings illustrates its limited value in these cases.

Lim et al. prospectively evaluated the performance of Anyplex™ plus against culture for respiratory specimens in a high-TB-burden setting and reported overall sensitivity of 87.5 and 69.2 % for smear-negative TB cases [21]. Perry et al. evaluated the performance of Anyplex™ plus against culture in a low-TB-burden setting in two arms; retrospective and prospective arms [22]. They reported sensitivity and specificity of 86 and 99 % respectively in the retrospective arm, and 100 and 96 % respectively in the prospective arm [22]. Both these studies were conducted in low HIV-burden setting and showed sensitivities higher than what we found. Despite novel technology in the assay and the improved extraction method utilized in our study, the assay had the lowest sensitivity.

Xpert® achieved 100 % specificity while MTBDR*plus* and Anyplex™ plus detected the presence of TB in culture-negative isolates. One isolate was positive on both assays and negative on Xpert®. Upon reviewing of laboratory records, this isolate was found to be from a patient with previous laboratory confirmed TB. The reason for the other false positive case detected only on MTBDR*plus* is unknown. False positive results on molecular assays have been attributed to the presence of non-viable organisms either due to patients being on treatment, thus, these assays are not recommended for monitoring response to therapy [23–25] or could also be due to inadequate buffering in the preparatory step for culture. Alternatively as these are open systems, laboratory cross-contamination is also a possibility [23]. Other reasons include a low mycobacterial load with concomitant harsh decontamination of the sample. Clinical interpretation would need to be assessed on a case to case basis reviewing previous diagnosis for TB as well as repeat testing.

There is no diagnostic test without flaws but molecular assays offer the potential for sensitive, specific and timely diagnosis. Xpert® offers in addition susceptibility to rifampicin which is used as a surrogate marker for multi-drug-resistant TB (MDR-TB) [12, 13]. This assay provides rapid results with minimal hands-on time and low potential for PCR contamination [12–14]. Unfortunately, the assay suffers a series of limitations; it requires an ambient temperature of <30 °C, storage space for the cartridges, uninterrupted supply of electric power which may not always be available in developing countries and the cost of waste disposal [14]. MTBDR*plus* has a potential risk of contamination through post-amplification processing and its interpretation of results is subjective if performed manually [11]. The interpretation of results is based on visual inspection of the strips and there can be inter-observer variation resulting in inconsistent reporting of results. We found MTBDR*plus* to be more labour-intensive as compared to Anyplex™ plus and Xpert®. Additionally, MTBDR*plus* and Anyplex™ plus require highly trained staff, sophisticated equipment and laboratory infrastructure. Anyplex™ plus offers rapid identification of non-mycobacterial tuberculosis (NTM) and may reduce the time and health-care cost associated with false-positive TB diagnosis based on smear microscopy. As mentioned earlier, Anyplex™ plus is an open two-step PCR assay and may increase chances of laboratory cross contamination. Anyplex™ plus may offer an alternative option particularly in low TB burden settings where very few cases of TB (less likely drug-resistant TB) are expected. In our study, we used the NucliSens® easyMAG® for extraction because we observed PCR inhibition when the manufacturer’s recommended method was followed. This resulted in additional cost to the assay. DNA extracted using the recommended method in MTBDR*plus* version 1.0 may be used on Anyplex™ plus as demonstrated by Perry et al. [22].

The limitation in our study is the use of stored residual specimens and we did not apply a final buffering step. Prior handling of the specimens as opposed to usage directly after decontamination might have compromised the quality of the specimens and thus the performance of the assays. However, we had directly compared the three assays which would not have been possible without testing from sediment. In addition, we used stored samples as we wanted to have an economical way of testing smear negative TB cases across all three systems, which are of importance in high HIV settings. An additional point to note is that our standard two stage laboratory sample processing applies centrifugation twice thus potentially enhancing the performance of the systems evaluated.

## Conclusion

Xpert® performed the best overall and was also superior in samples with low bacillary load as evidenced by its performance in TTP of 20 days or longer. The findings of our study supports the current WHO endorsement of Xpert® for smear negative TB. It is important that studies reporting on test performance stratify their results by TTP to accurately assess clinical performance especially in high HIV settings.

## Abbreviations

Anyplex™plus: Anyplex™ plus MTB/NTM/DR-TB Real-time detection
Ct: Threshold cycle
DNA: Deoxyribonucleic acid
DPO: Dual Priming Oligonucleotide
MTBDR*plus*: GenoTypeMTBDR*plus* Version 2.0
HIV: Human immunodeficiency virus
IUATLD: International Union Against Tuberculosis and Lung Disease
MDR-TB: Multi-drug-resistant TB
MGIT: Mycobacterial Growth Indicator Tube
MTBC: *Mycobacterium tuberculosis* complex
NHLS: National Health Laboratory Service
NTM: Non-tuberculous mycobacteria
PCR: Polymerase chain reaction
READ: Real Amplicon Detection
TTP: Time-to-positivity
TB: Tuberculosis
Xpert®: Xpert® MTB/RIF
WHO: World Health Organization
ZN: Ziehl-Neelsen
